# Implantation of a colorectal stent as a therapeutic approach in the treatment of esophageal leakage

**DOI:** 10.1186/1471-230X-7-10

**Published:** 2007-03-16

**Authors:** Jens-Gerd Scharf, Giuliano Ramadori, Heinz Becker, Annegret Müller

**Affiliations:** 1 Department of Gastroenterology and Endocrinology, Georg-August-University, Robert-Koch-Strasse 40, 37075 Göttingen, Germany; 2 Department of General Surgery, Georg-August-University, Robert-Koch-Strasse 40, 37075 Göttingen, Germany

## Abstract

**Background:**

While the mortality of esophageal surgery has decreased due to technological advancements, there is still a complication rate of about 30%. One of the main complications is the anastomotic leakage associated with a significant rate of morbidity and mortality. To close the leakage the efficacy of self-expanding stents (SES) has been shown in different studies. However, the high rate of stent migration limits the use of commercial available stents. In our case we were faced with the problem that the diameter of all available stents was too small to attach tightly to the mucosal wall of the esophagogastric anastomosis.

**Case presentation:**

We used, for the first time to our knowledge, a metal stent designed for colorectal application in an extensive anastomotic leak after esophageal resection in a patient with an esophageal cancer. After primary surgery with subtotal esohagectomy the anastomotic leak was stented endoscopically with a Polyflex self-expanding covered plastic stent after no response to intensive conventional management. Even though the stent was placed correctly, the diameter of the Polyflex stent was too small to attach onto the wall of the esophagogastric anastomosis. Again surgery was performed with a thoracal resection of the esophageal remnant and a hand made anastomosis. Unfortunately, again an anastomotic leak was detected soon after. To close the leak we decided to use a covered colorectal stent (Hanarostent) with an inner diameter of 30 mm. Sixteen weeks later the stent was extracted and complete mucosal healing of the esophageal leak was observed.

**Conclusion:**

The stent implantation with a large wide diameter offers a good chance to close more extensive leaks and prevent stent migration.

## Background

Although significant improvement in diagnosis and multimodal therapy of esophageal cancer has been made, this cancer entity is still associated with an unfavorable prognosis. Treatment options for esophageal cancer include surgery, chemotherapy and radiation, either individually or in combination. Patients with locally advanced esophageal cancer undergo combined radiochemotherapy with the aim to downsize the tumor followed by radical resection of the esophagus with curative intention. Progress in the field of operation techniques and the peri-operative management as well as treatment of patients in specialized medical centers decreased the mortality rate to about 3%. After radical surgery, the main factor limiting survival and quality of life is the frequency of acute postoperative complications. A study by Viklund et al. [1] revealed that the most common complications after esophageal resection were respiratory insufficiencies and serious infections. In 9% of patients (24 out of 275 patients) an anastomotic leakage was detectable. The rate of anastomotic leakage as a major complication was not depending on whether hand-sewn or stapled techniques were used, but were associated with the experience of the surgeon. Consequently, the occurrence of anastomotic leakage inversely correlated with the number of operations done by the surgeon [1].

Sealing of anastomotic leakages after esophageal resection, however, is the main field of endoscopic treatment procedures. Today, the placement of self expanding metal or plastic stents appears the best therapeutic option to treat anastomotic leakages [2,3]. Schubert et al. [4] reported complete closures of leakages after stent placement and stent removal later on in 11 out of 12 patients. Similarly, Hünerbein and colleagues [5] successfully implanted self-expanding plastic stents in 9 out of 9 cases with anastomotic leakage after esophagectomy. Using these plastic stents, complete sealing of the leak was reported in 7 out of 9 patients [6]. However, the main problem in the usage of the plastic stents was stent migration.

In the patient reported herein, we were confronted with the problem that the inner diameter of all commercial available plastic stents was too small to attach densely to the mucosal wall of the stomach, which has been used as substitute for the resected esophagus.

## Case presentation

**Figure 1:**
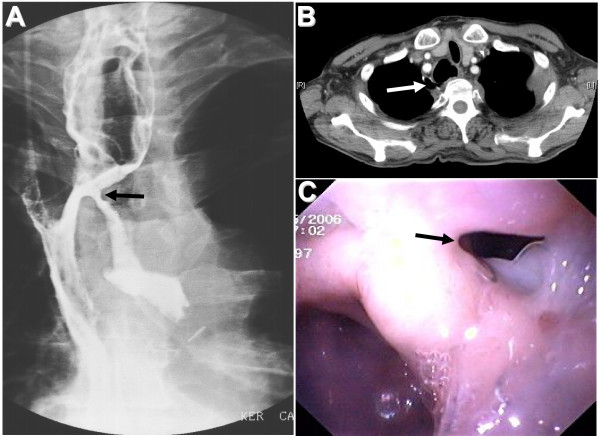
(A) Gastrografin swallow and (B) computed tomography showing the anastomotic leakage (arrows). (C) Endoscopic view of the same region.

We report on a 56 year old male patient diagnosed with an esophageal carcinoma located in the middle of the esophagus with a subtotal stenosis at 32 cm from the incisor teeth. Endosonography performed preoperatively revealed a locally advanced tumor (uT3, N+). Following the oncological recommendations the patient underwent neoadjuvant radiochemotherapy with a total of 50 Gy and a 5-fluorouracil (5-FU) based chemotherapy (5-FU 15 mg/kg body weight on days 1–5, cisplatin 75 mg/qm on day 7, repeated in week five). Thereafter, a subtotal esophageal resection combined with mobilization of the stomach through midline laparatomy and of the esophagus through the fifth intercostal area was done. The esophagogastric anastomosis was performed with a 25 mm stapler. Histological evaluation of the resected specimen revealed complete response of the esophageal carcinoma to the neoadjuvant radiochemotherapy with no tumor tissue detectable (ypT0, ypN0, ypMx). Increasing concentrations of acute-phase reactants in line with a dramatically decreasing general condition of the patient indicated a leakage that was confirmed by a gastrografin swallow demonstrating a anastomotic leak with gastrografin effusion into the left mediastinum (Fig. 1A). After lavage of the mediastinum and abdomen both were drained by a chest tube. Additionally, total parenteral nutrition as well as broad spectrum antibiotics were given. However, this nonoperative management to close the leak was unsuccessful and endoscopic stent implantation was planned.

**Figure 2:**
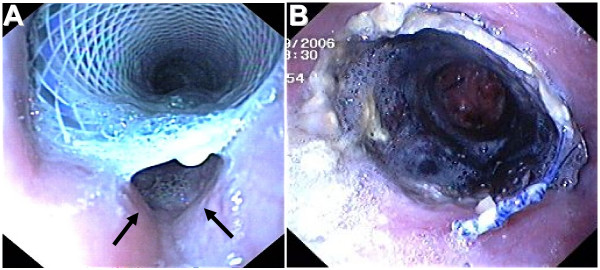
(A) Endoscopic view of the implanted Polyflex stent showing incomplete sealing of the esophagus lumen (arrows). (B) Complete closure of the anastomotic leakage was obtained by covered colorectal stent (Hanarostent).

The endoscopic examination showed a wide leak in the anastomosis located 25 cm from the incisor teeth (Fig. 1B). Because of the excellent prognosis of the patient, we initially used a Polyflex self-expanding covered plastic stent (Willy Rüsch GmbH, Kernen, Germany; stent dimensions: 25 mm flare with 21 mm body, length 120 mm) placed 2–3 cm distal the upper esophageal entrance to close the leakage (three weeks after the esophageal resection). Clinical change for the worse was suspicious of stent migration, but the endoscopic examination revealed that the polyflex stent was placed correctly. However, the caliber of 21 mm was undersized to attach tightly to the mucosa of the esophageal wall enabling running of fluids through the anastomotic leak (Fig. 2A). Without a commercial available esophageal stent with a larger inner diameter on hand a resection of the esophageal remnant and a hand made cervical esophageal anastomosis was performed six weeks after the first operation.

Unfortunately, a leak in the hand made anastomosis was detected again. To close the leak we decided to use for the first time a covered colorectal Hanarostent (MTW Endoskopie, Wesel, Germany) (stent dimensions: 30 mm body, length 60 mm) which was placed four weeks after the second operation. This stent is designed for colorectal applications. The unique structure of the membrane connects the several separated segments made of nitinol wire to increase the flexibility of the stent and to prevent migration and tumor growth.

**Figure 3:**
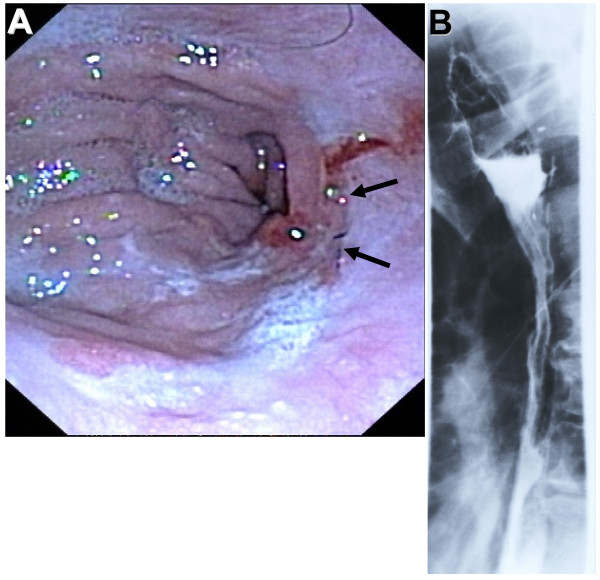
(A) Endoscopic view of the esophagogastric anastomosis demonstrating complete closure of the leakage after removal of the Hanarostent. Arrows indicate the area of the former leakage. (B) Gastrografin swallow.

This stent was placed without complications. Control endoscopy demonstrated a close seat of the stent to the gastric tube with complete covering of the anastomotic leak (Fig. 2B). Thereafter, the general condition of the patient increased continuously in line with a decrease of the acute-phase reactants. One week after metal stent implantation, oral feeding was restarted and the patient could be discharged from the hospital after another three weeks. Sixteen weeks after the second stent implantation, the stent was extracted and follow up endoscopy and gastrografin swallow indicated complete mucosal healing of the esophageal leak (Fig. 3).

## Conclusion

Esophageal resection is the standard procedure for the curative treatment of esophageal cancer. However, one major complication occurring after resection is the anastomotic leakage. In these cases, the endoscopic implantation of a stent is the most accepted treatment, especially in cases with a wide leackage. Different stents are available including self-expanding metal stents, self-expanding polyester stents and rigid plastic tubes which are placed endoscopically depending on the individual strategy for each patient. Due to complete response of the esophageal cancer to the neoadjuvant radiochemotherapy associated with a good prognosis of our patient, in an initial attempt a self-expanding plastic stent was placed over the anastomotic leakage. Plastic stents can easily be extracted after healing of the leakage whereas the main complication is stent migration observed in the range of 4.5 to 30 % [4,6-9]. After subtotal esophageal resection combined with mobilization of the stomach the migration rate is presumably even higher, because the distal end of the stent is free floating in the gastric lumen.

In our patient, the initial endoscopic treatment with a self-expanding Polyflex stent failed. This was due to the wide diameter of the anastomotic area which was not filled out by the self-expanding Polyflex stent pre-programming stent migration. This problem was solved by using a covered colorectal Hanarostent. This stent with a large shaft diameter enables a new strategy to close more extensive leaks and prevent stent migration. Stent implantation was followed by a rapid clinical improvement of our patient. According to the limited data available, self-expanding plastic stents apparently do not migrate more frequently than self-expanding metal stents [2]. However, it is more difficult to extract metal stents than plastic stents which should be performed before ingrowth of the stent. Due to impaired wound healing in our patient, we decided to extract the metal stent only 16 weeks after placement. Endoscopic removal of the Hanarostent was done without any major damage of anastomotic mucosa or any other complications. Endoscopic and radiological follow up examinations revealed complete healing of the anastomotic leakage. In summary, the covered colorectal Hanarostent with a large shaft diameter enables a new strategy to close more extensive anastomotic leaks and to prevent stent migration. In a curative setting, stent extraction after complete healing of the leakage is feasible.

## Competing interests

The author(s) declare that they have no competing interests.

## Authors' contributions

JGS, AM, enrolled the patient for clinical and surgical aspects, performed the endoscopic operations, drafting the article, read and approved the final manuscript

GR, critical revision of the article, read and approved the final manuscript

HB, performed surgical operations, critical revision of the article, read and approved the final manuscript

## Pre-publication history

The pre-publication history for this paper can be accessed here:



## References

[B1] Viklund P, Lindblad M, Lagergren J (2005). Influence of surgery-related factors on quality of life after esophageal or cardia cancer resection. World J Surg.

[B2] Costamagna G, Marchese M, Iacopini F (2006). Self-expanding stents in oesophageal cancer. Eur J Gastroenterol Hepatol.

[B3] Schubert D, Pross M, Nestler G, Ptok H, Scheidbach H, Fahlke J, Lippert H (2006). [Endoscopic treatment of mediastinal anastomotic leaks]. Zentralbl Chir.

[B4] Schubert D, Scheidbach H, Kuhn R, Wex C, Weiss G, Eder F, Lippert H, Pross M (2005). Endoscopic treatment of thoracic esophageal anastomotic leaks by using silicone-covered, self-expanding polyester stents. Gastrointest Endosc.

[B5] Hunerbein M, Stroszczynski C, Moesta KT, Schlag PM (2004). Treatment of thoracic anastomotic leaks after esophagectomy with self-expanding plastic stents. Ann Surg.

[B6] Gelbmann CM, Ratiu NL, Rath HC, Rogler G, Lock G, Scholmerich J, Kullmann F (2004). Use of self-expandable plastic stents for the treatment of esophageal perforations and symptomatic anastomotic leaks. Endoscopy.

[B7] Radecke K, Gerken G, Treichel U (2005). Impact of a self-expanding, plastic esophageal stent on various esophageal stenoses, fistulas, and leakages: a single-center experience in 39 patients. Gastrointest Endosc.

[B8] Radecke K, Lang H, Frilling A, Gerken G, Treichel U (2006). Successful sealing of benign esophageal leaks after temporary placement of a self-expanding plastic stent without fluoroscopic guidance. Z Gastroenterol.

[B9] Szegedi L, Gal I, Kosa I, Kiss GG (2006). Palliative treatment of esophageal carcinoma with self-expanding plastic stents: a report on 69 cases. Eur J Gastroenterol Hepatol.

